# DISPARITIES IN LOW DOSE COMPUTED TOMOGRAPHY LUNG CANCER SCREENING IN THE UNITED STATES: A MULTI-LEVEL ANALYSIS

**DOI:** 10.1093/geroni/igae098.4165

**Published:** 2024-12-31

**Authors:** Tung-Sung Tseng, Chien-Ching Li, Hui-Yi Lin, Kelsey N Witmeier, Yu-Wen Chiu, Michael Celestin, Edward J Trapido

**Affiliations:** LSUHSC, New Orleans, Louisiana, United States; Rush University, Chicago, Illinois, United States; Louisiana State University Health Sciences Center, New Orleans, Louisiana, United States; LSU Health Science Center New Orleans, New Orleans, Louisiana, United States; LSU Health Sciences Center, School of Public Health, Mew Orleans, Louisiana, United States; LSU Health New Orleans, New Orleans, Louisiana, United States; LSU Health Sciences Center, New Orleans, Louisiana, United States

## Abstract

Low-dose computed tomography (LDCT) screening is recommended for high-risk smokers to decrease lung cancer-related mortality and increase prognosis. In the U.S., the uptake of LDCT screening among eligible smokers is suboptimal. The impacts of social-environmental and individual factors on LDCT screening uptake using nationally representative datasets are understudied. The current study investigated multi-level factors associated with LDCT screening using national data.

**Methods:**

The 2017-2021 Behavioral Risk Factor Surveillance System (BRFSS) data, as well as other state-level variables (Medicaid expansion status and the number of screening facilities), were applied. The final study sample consisted of 15640 respondents from 29 states. All analyses were weighted to account for the complex sampling design applied in BRFSS.

**Results:**

The overall utilization rate of LDCT screening is only 18.4%. The LDCT screening rate varied by state (6.2 -31.1%). LDCT screen rates were positively associated with the number of lung cancer screening facilities per 10,000 smokers (r=0.67, p< 0.001). Among the respondents, individuals who were employed, never married, reported good health status, did not have primary care physicians, economic concerns like low income, did not have routine checkups, and did not have certain chronic conditions (i.e., cancer, asthma, COPD) had a lower utilization rate of LDCT screening compared with their counterpart.

**Conclusion:**

The use of LDCT screening among eligible smokers remains low. Enhancing access to care for high-risk individuals, promoting services to diverse racial and socioeconomic groups, and expanding Medicaid coverage to incorporate annual LDCT screening can be used to guide future lung cancer screening programs.

